# Inactivated *Lactobacillus plantarum* Carrying a Surface-Displayed Ag85B-ESAT-6 Fusion Antigen as a Booster Vaccine Against *Mycobacterium tuberculosis* Infection

**DOI:** 10.3389/fimmu.2019.01588

**Published:** 2019-07-09

**Authors:** Katarzyna Kuczkowska, Alastair Copland, Lise Øverland, Geir Mathiesen, Andy C. Tran, Mathew J. Paul, Vincent G. H. Eijsink, Rajko Reljic

**Affiliations:** ^1^Faculty of Chemistry, Biotechnology and Food Science, Norwegian University of Life Sciences, Ås, Norway; ^2^Institute for Infection and Immunity, St. George's University of London, London, United Kingdom; ^3^College of Medical and Dental Sciences, University of Birmingham, Birmingham, United Kingdom

**Keywords:** tuberculosis, vaccine, lactic acid bacteria, *Lactobacillus plantarum*, adjuvant, delivery vector

## Abstract

Vaccination is considered the most effective strategy for controlling tuberculosis (TB). The existing vaccine, the Bacille Calmette-Guérin (BCG), although partially protective, has a number of limitations. Therefore, there is a need for developing new TB vaccines and several strategies are currently exploited including the use of viral and bacterial delivery vectors. We have previously shown that *Lactobacillus plantarum* (*Lp*) producing Ag85B and ESAT-6 antigens fused to a dendritic cell-targeting peptide (referred to as *Lp*_DC) induced specific immune responses in mice. Here, we analyzed the ability of two *Lp*-based vaccines, *Lp*_DC and *Lp*_HBD (in which the DC-binding peptide was replaced by an HBD-domain directing the antigen to non-phagocytic cells) to activate antigen-presenting cells, induce specific immunity and protect mice from *Mycobacterium tuberculosis* infection. We tested two strategies: (i) *Lp* as BCG boosting vaccine (a heterologous regimen comprising parenteral BCG immunization followed by intranasal *Lp* boost), and (ii) *Lp* as primary vaccine (a homologous regimen including subcutaneous priming followed by intranasal boost). The results showed that both *Lp* constructs applied as a BCG boost induced specific cellular immunity, manifested in T cell proliferation, antigen-specific IFN-γ responses and multifunctional T cells phenotypes. More importantly, intranasal boost with *Lp*_DC or *Lp*_HBD enhanced protection offered by BCG, as shown by reduced *M. tuberculosis* counts in lungs. These findings suggest that *Lp* constructs could be developed as a potential mucosal vaccine platform against mycobacterial infections.

## Introduction

Tuberculosis (TB) continues to be one of the most deadly diseases in the world and has been designated as a global public health emergency by the World Health Organization (WHO) since 1993 (1). The WHO Global tuberculosis report of 2018 recounted 6.4 million new cases of TB in 2017 and the highest numbers of incidents were recorded in India, Indonesia and Nigeria (2). BCG, the only existing licensed TB vaccine, has been used in humans worldwide for nearly ten decades, although its efficacy remains debated (3–5). While BCG prevents TB infections in infants and children, the protection against pulmonary TB in adults and adolescents is incomplete and inconsistent (6, 7). Moreover, the BCG vaccine, though generally safe and well-tolerated, is inapplicable to immunosuppressed people, such as HIV-infected subjects, due to adverse effects and a risk of disseminated BCG infection (8–10). The questionable efficacy of BCG, together with increasing numbers of drug resistant strains of *Mycobacterium tuberculosis*, the causative agent of TB, raise an urgent need for developing a new effective vaccine that could halt the spread of TB.

*Mycobacterium tuberculosis* is a pathogen of the respiratory tract entering the body through mucosal tissue. Multiple studies have demonstrated that mucosal immunization to the airway (including intranasal or aerosol delivery), which mimics natural infections, is more protective than use of parenteral vaccines. It has been shown that mucosal delivery enhances the generation of tissue resident memory T cells that may inhibit the early phases of mycobacterial infection (11, 12).

A number of novel TB vaccine candidates are being considered and they include the following types: (i) prophylactic priming vaccines that may substitute BCG, (ii) prophylactic BCG boosting vaccines, and (iii) therapeutic vaccines for exposed individuals at risk of remission (4, 9). Currently, at least 13 vaccine candidates against TB are being tested in clinical trials (6, 9) including whole cell vaccines and subunit vaccines.

Today, viral vectors are the only heterologous vehicles for TB vaccines that have reached clinical trials. Vaccine candidates based on replication-deficient viruses include: (i) modified Vaccinia Ankara virus [MVA85A; (13, 14)], (ii) human adenovirus 5 [Ad5Ag85A; (15)], (iii) chimpanzee adenovirus [ChAdOx1.85A, (13)] or (iv) human influenza virus [TB/FLU-04L, (6, 9, 16)]. All of these carry antigen 85A, which is an enzyme involved in cell wall synthesis of *M. tuberculosis* (17). Besides viruses, bacteria-based heterologous vectors are attractive carriers for a potential new TB vaccine and studies in animal models have already shown encouraging results both for attenuated pathogens and non-pathogenic bacteria. For example, among pathogenic bacteria, attenuated *L. monocytogenes* expressing *M. tuberculosis* antigens enhanced protection against aerosolized *M. tuberculosis* in BCG-primed mice (18, 19). Similarly, *Salmonella typhimurium* secreting a mycobacterial antigen reduced numbers of tubercle bacilli in the lungs of infected mice (20, 21). While the utilization of attenuated pathogens in the TB vaccine field seems promising, this strategy poses a risk of virulence reversion, and therefore, there is an increasing interest in studying safer alternatives, such as lactic acid bacteria (LAB) or *Bacillus subtilis* spores. Interestingly, recombinant *B. subtilis* spores expressing TB antigens, were shown to be immunogenic (22), and more importantly, to induce protective immunity against aerosolized mycobacterial bacilli in a murine model (3, 23).

LAB are non-sporulating Gram-positive bacteria that, due to their safe status and well-developed genetic engineering toolbox, have been widely explored as vectors for delivery of prophylactic and therapeutic molecules for almost three decades (24–27). In vaccine development, *Lactococcus lactis* and *Lactobacillus* spp. are the most intensively explored, and *Lactobacillus planatrum* (25) and *L. lactis* (28–31) have been explored as carriers for DNA- (29–31) or protein-based (25, 28) TB vaccines. Although *L. lactis* remains the most utilized model LAB, *L. plantarum* (*Lp*) seems to be a more advantageous vaccine candidate due to its known immunomodulatory effects and adjuvant properties (32–37). In a recent study, we have shown that recombinant *Lp* producing a surface-anchored version of the *M. tuberculosis* Ag85B-ESAT-6 fusion protein induced *Mycobacterium*-specific immune responses when applied intranasally or orally in mice (25). The present study builds on these encouraging preliminary data in three ways: (1) construction of an alternative *Lactobacillus*-based vaccine strain, (2) in-depth and comparative analysis of induced immune responses, using a variety of experimental set-ups and, most importantly, and (3) assessment of the protective efficacy of the two vaccine candidates in an aerosolized low-dose TB model, using pathogenic *M. tuberculosis*. These experiments not only provide a first glimpse of the true potential of *Lactobacillus*-based vaccines in combatting TB, but also allow an evaluation of the extent to which immune responses observed in *in vitro* assays correlate with the protective potential of the vaccine candidate. Notably, protective efficacy of *Lactobacillus*-based TB vaccine candidates has not been reported previously.

We describe two candidates for a subunit vaccine against TB, based on *Lp* strains expressing a fusion of the Ag85B and ESAT-6 mycobacterial antigens, referred to as AgE6. AgE6 was expressed on the bacterial surface using an N-terminal lipoprotein anchor (25, 38) and a specific targeting molecule was fused to the antigen: a DC-binding peptide targeting bacteria to dendritic cells (construct *Lp*_DC) or a heparin binding domain (HBD) of mycobacterial heparin binding hemagglutinin (HBHA) that promotes binding to epithelial cells (construct *Lp*_HBD). The recombinant strains were subjected to *in vitro* phenotyping, followed by studies in mice to evaluate their protective potential against aerosolized *M. tuberculosis*. The study included two prime-boost immunization strategies: (i) a heterologous vaccination regimen where *Lp*-based vaccines were applied as a BCG booster, and (ii) homologous vaccination with *Lp*_DC or *Lp*_HBD.

## Materials and Methods

### Bacterial Strains, Plasmids, and Growth Conditions

The bacterial strains and plasmids used in this study are listed in Table 1. *Lactobacillus plantarum* constructs were cultured in MRS broth (Oxoid Ltd.) at 37°C without shaking. *Escherichia coli* TOP10 cells (Invitrogen) were grown in Brain Heart Infusion (BHI, Oxoid) broth at 37°C with shaking. Erythromycin was added to a final concentration of 10 μg/ml for *L. plantarum*. For *E. coli* final concentrations of erythromycin and ampicillin were 200 μg/ml and 100 μg/ml, respectively. Bacillus Calmette-Guérin Pasteur (BCG) and *M. tuberculosis* strain H37Rv were cultivated in 7H10 broth (Becton Dickinson) at 37°C. Liquid 7H10 was supplemented with ADC (Becton Dickinson), 0.05% Tween-80 and Selectab (Mast Diagnostics). Solid 7H11 medium was supplemented with OADC (Becton Dickinson), glycerol and Selectab (Mast Diagnostics). Liquid medium was solidified by adding 1.5% (w/v) agar.

**Table 1:** Plasmids and strains used in this study.

**Strain or plasmid**	**Description**	**References**
**PLASMIDS**		
pUC-AgE6	Amp^r^; pUC57 vector with synthetic *ag85besat6* gene	Genescript
pLp_1261AgE6-DC	Ery^r^; pLp_1261AgE6 (25) derivative, encoding the AgE6 antigen with a DC-binding peptide fused to its C-terminus	(25)
pEV	Ery^r^; control plasmid (“empty vector”)	(38)
pLp_1261AgE6-HBD	Ery^r^; pLp_1261AgE6-DC (25) derivative, encoding the AgE6 antigen with a heparin-binding domain (HBD) of HBHA fused to its C-terminus	This study
**STRAINS**		
*L. plantarum* WCFS1	Host strain	(39)
*E. coli* TOP10	Host strain	Invitrogen
*Lp_*DC	*Lp*_1261AgE6-DC*; L. plantarum* WCFS1 harboring pLp_1261AgE6-DC; using an N-terminal covalent lipoprotein anchor for surface display of the AgE6 antigen fused to DC-targeting peptide	(25)
*Lp_*HBD	*L. plantarum* WCFS1 harboring pLp_1261AgE6-HBD; using an N-terminal covalent lipoprotein anchor for surface display of the AgE6 antigen fused to HBD fragment	This study
*Lp*_Ev	*L. plantarum* WCFS1 carrying pEv (empty vector); used as a negative control strain	(38)
BCG	Bacillus Calmette-Guérin Pasteur strain	
*M. tuberculosis* H37Rv	Laboratory strain	

### DNA Manipulations and Plasmid Construction

**Figure 1:**
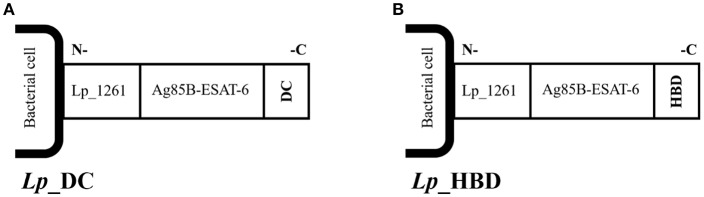
Schematic overview of the surface-anchored recombinant AgE6 fusion antigen. The N-terminal end of the recombinant AgE6 hybrid antigen was fused to the Lp_1261 lipoprotein anchor which attaches the protein to the cell membrane. Specific targeting peptides, here referred to as DC **(A)** or HBD **(B)**, were fused to the C-terminal end (i.e., the ESAT-6 side) of the AgE6 fusion antigen and thus form the most exposed part of the surface-anchored antigen. The DC-peptide targets dendritic cells, whereas the HBD fragment targets bacteria to epithelial cells.

The basic outline of the constructed expression vectors is shown in Figure 1. The fusion protein comprising the antigens Ag85B and ESAT-6 of *M. tuberculosis*, and referred to as AgE6, was designed as described previously (25). *Lp*_DC harbors the pLp_1261AgE6-DC plasmid that was constructed for inducible expression of the AgE6 antigen fused to DC-targeting peptide (40, 41) and anchored to the bacterial surface via the Lp_1261 lipoprotein anchor, as described in a previous study (25). In order to engineer *Lp*_HBD construct, the *lp1261age6hbd* gene fragment was designed such as to fuse a 147 bp fragment encoding the heparin-binding domain (referred here to as HBD) from *M. tuberculosis* heparin-binding hemagglutinin (HBHA; GenBank: AAC26052.1) to the C-terminal end of AgE6. Three-amino acid linker, encoding Gly-Thr-Ala and containing a *KpnI* restriction site, was introduced between AgE6 and HBD. *NdeI* and *HindIII* restriction sites were introduced upstream and downstream the *lp1261age6hbha* gene fragment, respectively. Such designed *lp1261age6hbha* DNA fragment was codon optimized for expression in *L. plantarum*, synthesized at Genscript (Piscataway), and cloned into a pUC57 plasmid, yielding pUC-HBD. The pUC -HBD plasmid was digested by *NdeI* and *HindIII*. The resulting 1560 bp Lp_1261-AgE6-HBD-encoding fragment was cloned into *NdeI/HindIII* digested pLp_1261AgE6-DC (25), yielding pLp_1261AgE6-HBD. The resulting plasmid was first transformed into *E. coli* TOP10. Positive clones were screened by PCR and restriction enzyme digestion after which the PCR-amplified fragments were verified by sequencing using primers SekF (GGCTTTTATAATATGAGATAATGCCGAC) and SekR (CCTTATGGGATTTATCTTCCTTATTCTC). Plasmid pLp_1261AgE6-HBHA was purified using PureYield^TM^ Plasmid Miniprep System (Promega Corporation) and electroporated into *L. plantarum* cells according to Aukrust et al. (42), yielding *Lp*_HBD construct.

### Preparation of *L. plantarum* Constructs

The expression of recombinant protein was induced and bacterial cells were harvested 3 h after induction as described elsewhere (25, 43). Recombinant bacteria were inactivated by UV-irradiation for 1 h and the successful inactivation was confirmed. Pellets of inactivated bacteria were stored at −80°C until use. In order to determine the number of CFU, freshly harvested bacterial cells were cultivated on solid MRS medium supplemented with antibiotics for 48 h and the colonies were counted. The numbers of inactivated bacteria were verified after storage at −80°C by counting in a Buerker counting chamber (Paul Marienfeld GmbH & Co. KG).

### Growing of *Mycobacterium* Strains

Bacillus Calmette-Guérin Pasteur and *M. tuberculosis* were grown to log phase at 37°C in complete liquid 7H10 broth, harvested and cryopreserved in liquid nitrogen until use. Freshly harvested mycobacterial cells were cultivated on complete 7H11 agar plates for 3-4 weeks and the CFU were determined.

### Western Blot Analysis

To analyze expression of recombinant fusion antigen in *L. plantarum*, cells from a 50 mL culture were harvested 3 h after induction (25, 43) and resuspended in 500 μL PBS. Bacterial protein extracts were prepared by disruption in FastPrep tubes containing 1.5 g of glass beads (size ≤ 106 μm; Sigma-Aldrich), using a FastPrep® FP120 Cell Disrupter with a shaking speed of 6.5 m/s for 45 s. After 5 min incubation on ice the shaking process was repeated. The glass beads were removed by sedimentation and the protein extracts were transferred to a new tube. Proteins were separated by SDS-polyacrylamide gel electrophoresis using 10% Mini-Protean TGX Precast gels (BioRad) and transferred to a nitrocellulose membrane using the iBlot^TM^ Dry Blotting System (Invitrogen). The proteins were detected using the SNAP i.d.® 2.0 Protein Detection System (Merck) using a specific monoclonal mouse anti-ESAT-6 antibody (Abcam) diluted 1:15000 and, subsequently, a polyclonal HRP-conjugated rabbit anti-mouse IgG (DAKO), diluted 1:7500. Proteins were visualized using the SuperSignal™ West Pico Chemiluminescent Substrate (Termo Fisher Scientific) and signals were documented using an Azure c400 system and AzureSpot Analysis Software (Azure Biosystems), following the manufacturer's instructions.

### Flow Cytometry and Indirect Immunofluorescence Microscopy of *L. plantarum* Expressing Recombinant AgE6 Antigen

Surface display of recombinant AgE6 was analyzed in UV-inactivated *L. plantarum* cells. Approximately 1 × 10^9^ bacterial cells were probed with monoclonal mouse anti-ESAT-6 antibody, followed by incubation with polyclonal rabbit FITC-conjugated anti-mouse antibody, as described previously (25). Stained bacterial cells were analyzed by flow cytometry using a MACSQuant analyzer (Miltenyi Biotec), following the manufacturer's instructions. For indirect immunofluorescence microscopy, bacteria were visualized on a Zeiss Axio Observer.z1 microscope (Zeiss) and the fluorescence was acquired by EX 450-490 nm and EM 500-590 nm (EX, excitation; EM, emission).

### Isolation of Human Dendritic Cells (DCs)

Human peripheral blood mononuclear cells were isolated and handled according to institutional ethical guidelines (Østfold Hospital Trust, Norway), as described elsewhere (25). Briefly, cells were isolated by density gradient centrifugation for 25 min at 1,500 × *g* using Lymphoprep TM (Axis-Shield Diagnostics Ltd.) at room temperature and washed four times with PBS to remove the platelets. CD14^+^ cells were separated on an LS column (Miltenyi Biotec) using human CD14 MicroBeads (Miltenyi Biotec). Cells were seeded in 24-well plates (1 × 10^6^ cells/well) and maintained in complete RPMI medium (RPMI supplemented with 10% fetal bovine serum FBS, 1% penicillin streptomycin, 2 mM l-glutamine, and 50 μM 2-mercaptoethanol, all from Sigma-Aldrich) with 25 ng/mL rhIL-4 and 50 ng/ml rhGM-CSF (ImmunoTools) for 4 days followed by replacement with fresh IL-4- and GM-CSF-supplemented complete medium and cultivation for another 3 days.

### Cell Lines

The mouse macrophage cell line J774.2 was cultured in complete DMEM medium (DMEM supplemented with 10% fetal bovine serum FBS, 1% penicillin-streptomycin, 2 mM l-glutamine, and 50 μM 2-mercaptoethanol, all from Sigma-Aldrich). Cells were maintained in a humidified incubator at 37°C and 5% CO_2_.

### Isolation of Bone Marrow-Derived Dendritic Cells (BMDCs)

Bone marrow-derived dendritic cells (BMDCs) were isolated as described before (3, 44). Briefly, freshly isolated myeloid cells were incubated in complete RMPI supplemented with 20 ng/mL murine GM-CSF for 7 days. Non-adherent cells (including lymphocytes and granulocytes) were removed at days 2 and 4 followed by adding fresh medium supplemented with GM-CSF. Proportions of CD11c+ cells above 80% were routinely obtained. The cells were maintained in complete RPMI medium in a humidified incubator at 37°C and 5% CO_2_.

### Flow Cytometry of Human DCs

Cells were pre-incubated with human FcR Blocking Reagent (Miltenyi Biotec) diluted 1:50 in flow cytometry buffer containing 0.5% BSA (Sigma-Aldrich) and 2 mM EDTA in order to block non-specific binding of immunoglobulins to the Fc receptors. The cells were then stained with specific anti-human antibodies for 20 min at 4°C and analyzed by flow cytometry using a MACSQuant analyzer and the data were processed using FlowJo software (FlowJo LLC).

### Flow Cytometry of Mouse Cells

Cells were pre-incubated in PBS containing TruStain fcX™ Fc Receptor Blocking Solution (BioLegend) diluted 1:500, and eBioscience™ Fixable Viability Dye eFluor™ 780 diluted 1:1000 in order to block non-spiecific binding of immunoglobulins to the Fc receptors and exclude dead cells, respectively. Subsequently, cells were stained with specific anti-mouse antibodies for 45 min at 4°C. For intracellular staining of cytokines, the cells were fixated in BD Cytofix™ Fixation Buffer (BD Biosciences) prior to staining and 0.5% saponin (Sigma-Aldrich) was included in the flow cytometry buffer. After staining, cells were analyzed on FACSCanto II flow cytometer (BD Biosciences) and the data were analyzed and processed using FlowJo software.

### Activation of Antigen-Presenting Cells (APCs)

Human DCs (1 × 10^6^) were stimulated with *L. plantarum* at a multiplicity of infection (MOI) of 200. A cocktail of 100 ng/ml LPS, 15 ng/ml TNF-α (ImmunoTools) and 5 μM PGE2 (Sigma-Aldrich) was included as a positive control. After 48 h incubation, the cells were detached with trypsin (Biowest) and transferred to a V-bottom 96-well plate. Subsequently, the human DCs were stained with specific anti-human antibodies for cell surface molecules: VioBright FITC-conjugated CD40 diluted 1:50, PE-conjugated CD80 diluted 1:11, APC-conjugated CD86 diluted 1:11 and PE-conjugated HLA-DR diluted 1:11 (all from Miltenyi Biotec).

Murine macrophages or DCs (2 × 10^5^) were seeded in triplicate in 96-well plates and allowed to adhere to the well surface for 2–4 h in a humidified incubator at 37°C and 5% CO_2._ The cells were stimulated with recombinant *L. plantarum* at an MOI of 100 or with 100 ng/mL LPS for 24 h or 48 h. The cells were then stained with specific anti-mouse antibodies for cell surface molecules: PerCP/Cy5.5-conjugated CCR7, Brilliant Violet 510™-conjugated MHC class II, FITC-conjugated MHC class I, Brilliant Violet 421™-conjugated PD-L1, PE-conjugated PD-L2, APC-conjugated CD80 and PE/Cy7-conjugated CD86 (all from BioLegend), all diluted 1:150.

### Cytokine Analysis

The pro-inflammatory cytokines were analyzed by ELISA in culture supernatants from stimulated APCs. Murine IL-6, TNF-α, IL-1β, and IFN-γ were measured by ELISA using eBioscience Ready-Set-Go kits following the manufacturer's instructions and plates were read on a Tecan200 plate-reader. Human IL-6, TNF-α, IL-1β, and IL-12 were measured using PeproTech ABTS ELISA Development Kits following the manufacturer's instructions and plates were read on a Sunrise Plate Reader (Tecan). IL-12p40 was detected by intracellular cytokine staining after 24 h stimulation of murine APCs in the presence of 10 μg/mL brefeldin A (Sigma-Aldrich) using IL-12p40-PE (BioLegend) diluted 1:50.

### Animals

Female C57BL/6 mice at the age of 6–8 weeks were obtained from Charles River, UK and were divided in 6 groups (*n* = 10). All animals were used with approval from St. George's University of London Ethics Committee under an approved UK Home Office animal project license and used in accordance with the Animals (Scientific Procedures) Act 1986.

### Vaccines

BCG, *Lp*_DC and *Lp*_HBD cultures were prepared as described above. For *Lp* vaccines, the immunization dose consisted of 1 × 10^9^ bacterial cells and 20 μg the adjuvant poly (I:C) (Sigma-Aldrich) in sterile PBS. For BCG vaccine, the immunization dose contained 5 × 10^5^ CFU BCG Pasteur in sterile PBS. Mice were administered with 100 μL and 50 μL for subcutaneous and intranasal immunization, respectively. All intranasal inoculations were performed under light anesthesia using isoflurane.

### Immunization Protocol

**Figure 2:**
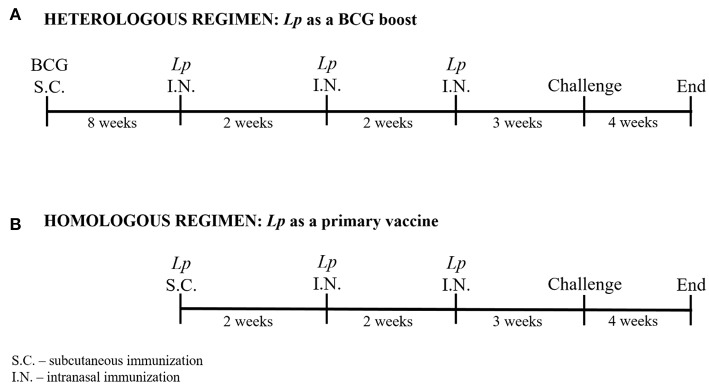
Immunization protocols. Two prime-boost immunization strategies were applied: a heterologous regimen including parenteral BCG immunization followed by three intranasal *Lp* boosts **(A)**, and a homologous regimen including parenteral *Lp* immunization followed by two intranasal boosts **(B)**. Mice were challenged with *M. tuberculosis* via aerosol delivery 3 weeks after the last vaccination. The experiment was terminated 4 weeks after mycobacterial infection.

Two prime-boost immunization regimens were applied to test *Lp* vaccines efficacy: i) a heterologous regimen where *Lp* constructs were tested as a booster to BCG vaccine, and ii) a homologous regimen where *Lp* constructs were used as a primary vaccine. The schematic protocol is presented in Figure 2.

#### Heterologous Regimen (Figure 2A)

Mice were immunized subcutaneously with BCG followed by intranasal boosting with *Lp* vaccines given in three doses administered every 2 weeks, starting 8 weeks after the BCG priming.

#### Homologous Regimen (Figure 2B)

*Lactobacillus plantarum* vaccines were given subcutaneously followed by two intranasal applications given every 2 weeks after priming.

Three weeks after the last immunization, three animals from each group were sacrificed for immunogenicity analysis and the remaining animals were used in a *M. tuberculosis* challenge experiment.

### Challenge With *M. tuberculosis*

Mice were infected with ~200 *M. tuberculosis* bacilli per animal delivered via low-dose aerosol, using a Biaera aerosol generator (Biaera Technologies). Four weeks after the infection, the mice were sacrificed and lungs and spleens were taken in order to evaluate mycobacterial burden. The organs were homogenized in a stomacher (Seward) containing 0.1% Triton X-100 and the homogenates were plated in duplicates (lungs) or singlets (spleens) on completed 7H11 agar and incubated at 37°C for 3-4 weeks. Subsequently, CFU were counted.

### Preparation of Tissues From Mice

#### Isolation of Splenocytes

The spleens were collected, mashed through 70 μm Corning® cell strainers (Sigma-Aldrich) and centrifuged at 300 × *g* for 10 min at room temperature. The cell pellets were resuspended and incubated in Red Cell Lysis buffer (Sigma-Aldrich) for 5 min, recovered by centrifugation and washed with RPMI 1640 medium. Cells were maintained in complete RPMI medium in a humidified incubator at 37°C and 5% CO2.

#### Serum Preparation

Blood collected from mice was allowed to clot at room temperature for 1 h, followed by centrifugation at 1,000–2,000 × *g* for 10 min at 4°C. Serum was stored at −20°C until analysis.

#### Bronchoalveolar Lavage (BAL)

One milliliter of sterile PBS was injected into the lungs via trachea and following three rounds of flashing the washes were collected and stored at −20°C until analysis.

### IgG and IgA Antibodies Assays

ELISA assays were used to determine antigen-specific titers for IgG in serum and IgA in BAL. Microtiter plates were coated with 5 μg/ml Ag85B or ESAT-6 (both from Lionex GmbH) and incubated overnight at room temperature followed by blocking with 1% BSA (Sigma-Aldrich) in PBS for 1 h at 37°C. Serial dilutions of serum (10-fold) and BAL (3-fold) samples were applied to the pre-coated plates followed by incubation for 1 h at 37°C. Subsequently, the plates were incubated for 1 h at 37°C with HRP-conjugated anti-mouse IgA or IgG antibodies (Sigma-Aldrich), both diluted 1:1,000, for detection of IgA or IgG, respectively. OPD Substrate Tablets (Sigma-Aldrich) were used for color development, following the manufacturer's instructions. The OD at 450 nm was measured after 15 min incubation at room temperature.

### T Cell Proliferation

Freshly isolated splenocytes (1 × 10^E6^) were seeded in 96-well plates and stimulated with 5 μg/mL Ag85B antigen or 1 μ/mL ESAT-6 antigen. As positive control, the cells were stimulated with 1 μg/mL α-CD3 (BioLegends). Antigen-specific T cell proliferation was analyzed after 6 days of incubation with antigens. The cells were blocked, as described above, and subsequently stained with PerCP/Cy5.5-conjugated CD4, Brilliant Violet 510™-conjugated CD8a, FITC-conjugated CD44, PE-conjugated CD62L and Brilliant Violet 421™- conjugated CD90.2 antibodies (all from BioLegends), all diluted 1:100. After staining, the cells were fixed using eBioscience™ Foxp3/Transcription Factor Staining Buffer Set and permeabilized using eBioscience™ Permeabilization Buffer, according to the manufacturer's instructions. Subsequently, cells were intracellularly stained with 1:50 diluted APC-conjugated Ki-67 antibody (BioLegends) and analyzed by flow cytometry.

### T Cell Polyfunctionality

In order to analyze multifunctional antigen-specific T cells, freshly isolated splenocytes (1.5 × 10^6^) were seeded in 96-well plates and stimulated with 5 μg/mL Ag85B or 1 μ/mL ESAT-6 antigens. As a positive control, cells were stimulated with 100 ng/mL PMA and 1 μg/mL Ionomycin. After 4 h incubation, cells were blocked and fixed, as described in *Flow cytometry of mouse cells* paragraph. Subsequently, cells were stained with FITC-conjugated CD3, PerCP/Cy5.5-conjugated CD4, Brilliant Violet 510™-conjugated CD8a, APC-conjugated TNF-α, PE/Cy7-conjugated IL-17A, PE/Cy5-conjugated IL-2 and Brilliant Violet 421™-conjugated IFN-γ antibodies (all from BioLegends), all diluted 1:100, and analyzed by flow cytometry.

## Results

### The *Lp* Constructs Display the AgE6 Antigen on the Bacterial Surface

The AgE6 hybrid antigen was displayed on the bacterial surface through an N-terminal lipoprotein anchor derived from Lp_1261, which ensured covalent biding of the antigen to the bacterial cell membrane (25, 38). Two targeting peptides were selected in order to target AgE6-producing bacteria to eukaryotic cells and a schematic overview of the constructs is shown on Figure 1. The *Lp*_DC construct was constructed as described previously (25) so that the DC-targeting peptide was fused to the C-terminal end of the AgE6 antigen (Figure 1A). In the *Lp*_HBD construct, the HBD fragment from the HBHA surface protein of *M. tuberculosis* was fused to the C-terminal end of the AgE6 antigen (Figure 1B) replacing the DC-targeting peptide. HBD is a methylated lysine-rich C-terminal fragment of HBHA that mediates an interaction with epithelial and non-phagocytic cells (45–47).

**Figure 3:**
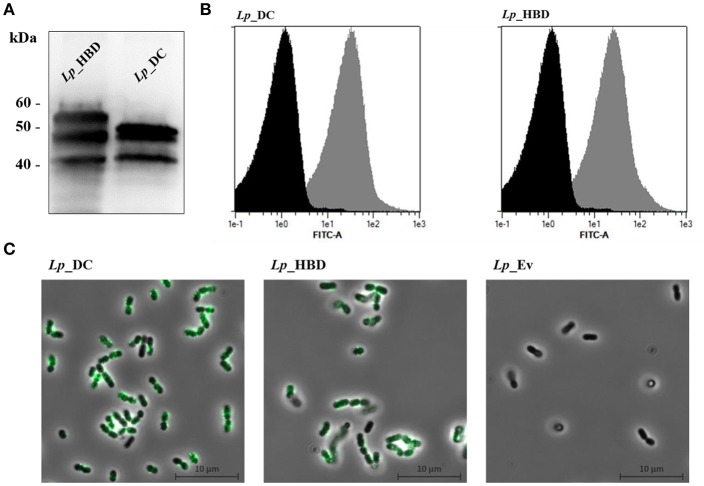
Production **(A)** and surface localization **(B,C)** of the recombinant AgE6 antigen. Production of the anchor-fused AgE6 in *Lp*_DC and *Lp*_HBD constructs was analyzed by Western blotting and is shown in **(A)**. The positions of size marker proteins are indicated and predicted sizes of the detected proteins (Figure 1) are 48.4 and 54.1 kDa for *Lp*_DC and *Lp*_HBD constructs, respectively. The presence of AgE6 on the bacterial surface was detected using immunofluorescent methods: flow cytometry and fluorescent microscopy. *Lp*_Ev was used as a negative control strain. Panel **B** shows flow cytometry analysis for *Lp*_DC and *Lp*_HBD (gray histograms) and *Lp*_Ev (black histograms). Panel C shows indirect immunofluorescence microscopy of the indicated constructs. The data presented are from a representative experiment. Each experiment was performed three times, giving similar results.

The expression of recombinant AgE6 antigens was evaluated by Western blot analyses of cell-free crude protein extracts from growing and induced *L. plantarum* constructs. Figure 3A shows bands of the correct size for both *Lp*_DC (48.4 kDa) and *Lp*_HBD (54.13 kDa), as well as some smaller protein fragments that are likely degradation products, while no bands were detected in a protein extract of the negative control strain (data not shown). Flow cytometry of bacteria labeled with an anti-ESAT-6 antibody showed drastically increased fluorescence intensity for both antigen-producing constructs when compared to the negative control strain, *Lp*_Ev (Figure 3B). Fluorescent microscopy gave clear signals for bacteria expected to produce AgE6 but not for *Lp*_Ev (Figure 3C).

### The *Lp* Constructs Activate Human and Mouse APCs

**Figure 4:**
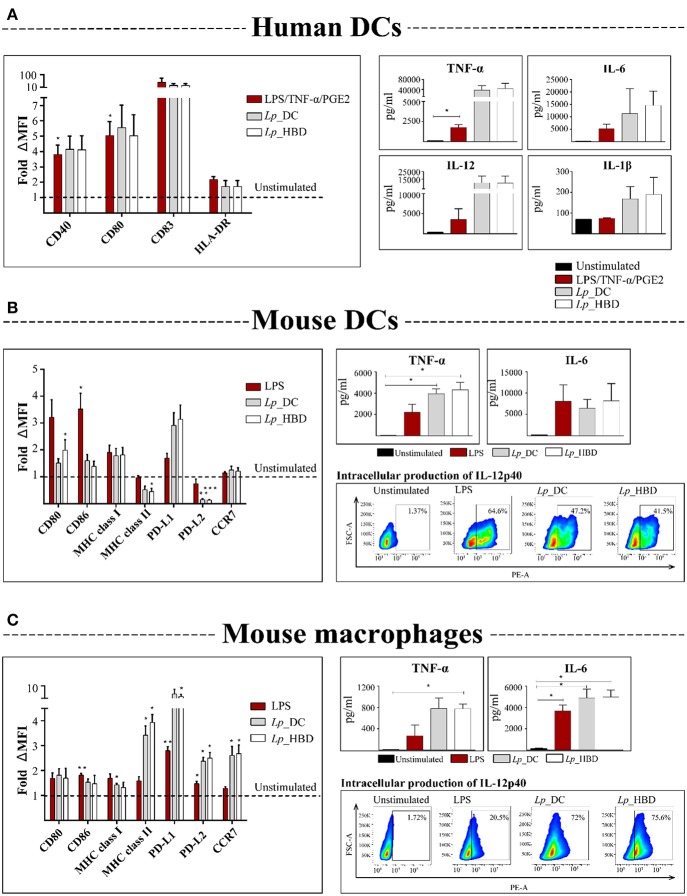
Activation of human DCs **(A)**, mouse DCs **(B)**, and mouse macrophages **(C)** by UV-inactivated lactobacilli. APCs were incubated with *Lp*_DC or *Lp*_HBD at MOI of 200 for human cells and 100 for mouse cells, for 24 or 48 h. Expression of surface co-stimulatory molecules was measured by flow cytometry and the median fluorescence intensity (MFI) was normalized to the unstimulated controls. Pro-inflammatory cytokines in culture supernatants were quantified by ELISA. IL-12p40 in mouse APCs was detected by intracellular staining followed by flow cytometry and the plots are from a representative experiment of cells stimulated with LPS (100 ng/mL), *Lp*_DC or *Lp*_HBD. For human DCs **(A)**, three blood donors were tested and the data are shown as a mean ± SEM. The results for mouse cells **(B,C)** are presented as a mean from three independent experiments and the data are shown as a mean ± SEM. Statistically significant differences were tested against the unstimulated controls using paired *t*-test and are indicated as follows: *p < 0.05, **p < 0.01, and ***p < 0.001.

Activation of professional APCs, such as dendritic cells and macrophages, is essential for post-immunization T cell priming (3, 48). Conserved bacterial components, so called microbe- or pathogen-associated molecular patterns (MAMPs or PAMPs, respectively), can contribute to APCs activation reflecting in expression of co-stimulatory molecules and secretion of cytokines. We investigated the ability of the *Lp*_DC and *Lp*_HBD vaccine candidates to activate human DCs and mouse DCs and macrophages. The innate immune cells were stimulated with UV-inactivated bacteria for 48 h and examined for upregulation of co-stimulatory molecules and production of pro-inflammatory cytokines (Figure 4).

The results for human DCs showed a tendency toward upregulation of CD83, CD40, CD80, and HLA-DR molecules, and showed elevated concentrations of secreted IL-6, IL-12, TNF-α, IL-1β, after stimulation with both *Lp* constructs (Figure 4A). Notably, the levels of measured cytokines were higher for *Lp*-stimulated DCs, compared to LPS/TNF-α/PGE2 stimulation, which was used as positive control, although the difference was not statistically significant (Figure 4A).

The assessed expression of surface markers in murine DCs revealed that *Lp*_HBD upregulated CD80 (*p* < 0.05) (Figure 4B). For both *Lp*_DC and *Lp*_HBD a tendency toward upregulation of PD-L1, MHC class I and, in lesser degree, CD86, was observed, but differences were not statistically significant (Figure 4B). None or very low upregulation was detected for CCR7, while MHC class II and PD-L2 were downregulated (Figure 4B). Quantification of secreted pro-inflammatory cytokines showed high production of TNF-α (*p* < 0.05) in cells exposed to *Lp*_DC or *Lp*_HBD and a tendency toward elevated IL-6 levels (Figure 4B).

For mouse macrophages, we detected significant upregulation of MHC class I, MHC class II, PD-L1, PD-L2, and CCR7 upon exposure to each of the two *Lp* constructs and tendencies toward up-regulation of CD80 and CD86. Cytokine measurements revealed significantly increased concentrations of TNF-α (*p* < 0.05 for *Lp*_HBD) and IL-6 (*p* < 0.05) as shown in Figure 4C.

Production of IL-1β was not detected in the supernatants from mouse APCs (data not shown). Analysis of intracellularly produced and captured IL-12p40 in mouse immune cells indicated positive responses to LPS, *Lp*_DC and *Lp*_HBD (Figures 4B,C).

Thus, *Lp*_DC and *Lp*_HBD activated APCs and induced multiple cytokine responses, but we did not observe any significant differences between the two constructs in *in vitro* assays.

### *Lp*_DC and *Lp*_HBD Induce Moderate Antibody Responses

We tested the *Lp*-based vaccine candidates in a mouse model using two different immunization regimens as outlined in Figure 2, and included non-immunized mice (naïve) and single parenteral BCG immunization as controls. Three weeks after the last vaccination, immune response were assessed.

**Figure 5:**
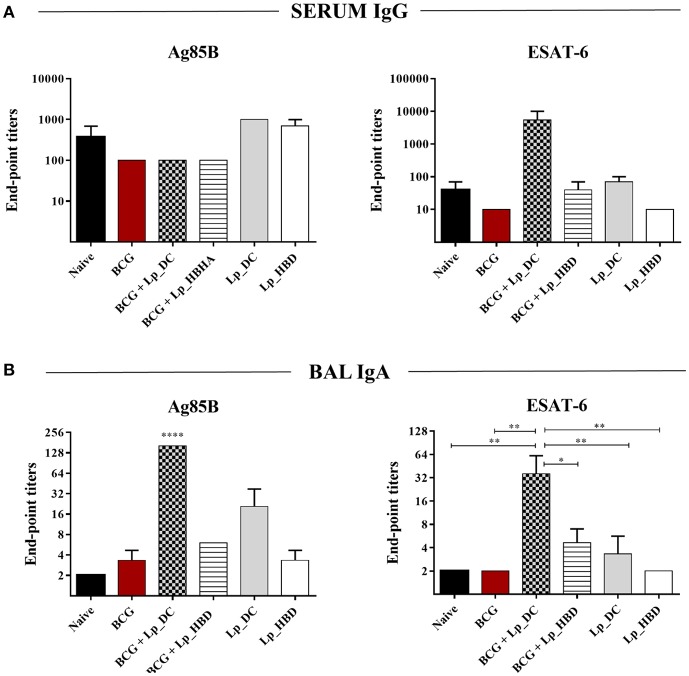
Humoral responses induced by *Lp*_DC and *Lp*_HBD. Serial dilutions of serum (10-fold) and BAL (3-fold) samples were subjected to ELISA in order to determine antibodies specific to Ag85B or ESAT-6. The end-point titers were evaluated for serum IgG **(A)** and BAL IgA **(B)**. Results are presented as a mean ± SEM (*n* = 2–3). Statistically significant differences were determined using one-way ANOVA with Tukey's *post-hoc* test and are indicated as follows: *p < 0.05, **p < 0.01, and ****p < 0.0001.

In order to evaluate humoral responses, we measured the titers of antigen-specific IgG in serum samples and IgA in lung washes, specific to Ag85B or ESAT-6 antigens. Analysis of serum samples showed no significant elevation of anti-Ag85B IgG in any of the groups but showed a tendency toward increased levels of IgG against ESAT-6 upon heterologous immunization with *Lp*_DC (Figure 5A). Studies of lung washes showed significantly improved IgA titers for mice boosted with *Lp*_DC in the heterologous regimen for both Ag85B and ESAT-6 and also a noticeable increased anti-Ag85B IgA in mice that received *Lp*_DC as a primary vaccine (Figure 5B). *Lp*_HBD induced modest increase of IgA specific to both antigens in lung washes only when given as a BCG boost (Figure 5B).

Taken together, the results suggest that *Lp*-based constructs elicit merely low or moderate levels of antibody responses to the antigens, both systemically and in the mucosa, and in general the effects were better detectable for *Lp*_DC construct.

### *Lp*_DC and *Lp*_HBD Enhance BCG-Induced T Cell Proliferative Responses

**Figure 6:**
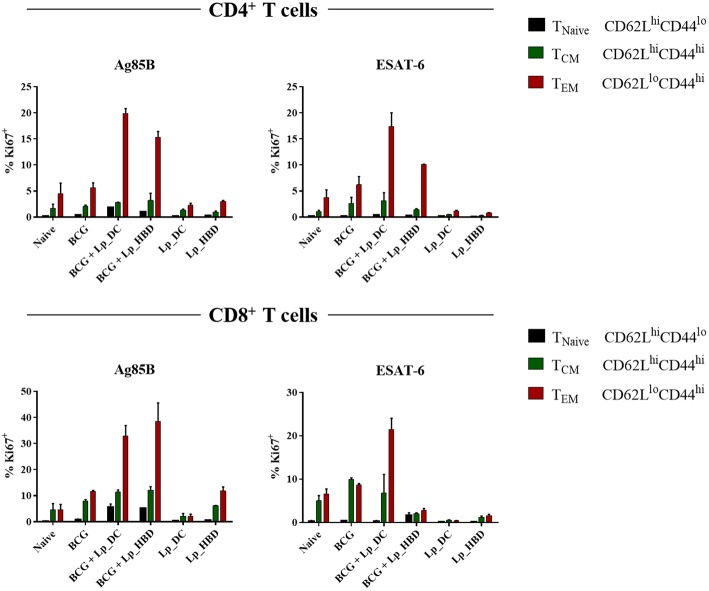
Antigen specific T cell proliferation. Splenocytes from immunized mice were stimulated with Ag85B or ESAT-6 for 6 days. T cell proliferation was analyzed by Ki67 staining using a live → single cells → CD3^+^ → CD4^+^/CD8^+^ → Ki67^+^ gating strategy. Central memory T cells (T_CM_), effector memory T cells (T_EM_) and naive T cells (T_Naive_) were determined using CD44 and CD62L staining. Results are presented as a mean ± SEM of technical duplicates and are derived from *n* = 2–3 pooled spleens per group.

In the next steps, we investigated the cellular immunity evoked by the *Lp*-based vaccine candidates. First, we examined whether immunization with *Lp*_DC or *Lp*_HBD induced antigen-specific T cell proliferation in the spleen. To do so, we examined expression of proliferation marker Ki67 in splenocytes from immunized mice by incubating with recalling antigens (Figure 6). Furthermore, using the CD44 and CD62L markers, we discriminated between T cell phenotypes: T central (T_CM_; CD44^hi^CD62L^hi^), T effector (T_EM_; CD44^hi^CD62L^lo^) memory cells, and naïve T cells (T_Naive_ CD44^lo^CD62L^hi^) (Figure 6). Among Ag85B-stimulated splenic CD4^+^ T cell populations, we observed increased levels of Ki67^+^ cells in groups boosted with *Lp*_DC (~25%, for all three phenotypes together) or *Lp*_HBD (~19%) in the heterologous regimen, compared to non-immunized mice (~6%) and to the BCG only group (~8%). The populations of CD8^+^ T cells showed elevated numbers of Ag85B-induced Ki67^+^ T cells in mice singly immunized with BCG (~20%) and high proliferative responses for groups of BCG-primed mice that had been boosted with *Lp*_DC (~50% Ki67^+^) or *Lp*_HBD (~56% Ki67^+^), compared to naïve mice (~9% Ki67^+^).

Stimulation with the ESAT-6 antigen revealed only marginally elevated levels of proliferating CD4^+^ T cells in mice that had received only BCG (~9% Ki67^+^), but stronger responses in mice given heterologous boosts with *Lp*_DC (~21% Ki67^+^) or *Lp*_HBD (~12% Ki67^+^). Ki67^+^CD8^+^ populations were increased for mice immunized singly with BCG (~19%) and boosted with Lp_*DC* (~28%), but not with *Lp*_HBD (~12%).

Of note, antigen specific T-cell proliferation was not detected for mice subjected to the homologous regimen primarily immunized with *Lp*-based vaccines. Thus, taken together, the overall picture emerging from Figure 6 is that T cell proliferation for mice immunized with BCG and mucosally boosted with *Lp*_DC or *Lp*_HBD was considerably higher compared to BCG alone or to the homologous prime-boost regimen. The populations of proliferating CD4^+^ and CD8^+^ T cells were dominated by the T_EM_ phenotype, whereas proportion of the T_CM_ phenotype remained modest (Figure 6).

### *Lp*_DC and *Lp*_HBD Induce Antigen-Specific IFN-γ Secretion

**Figure 7:**
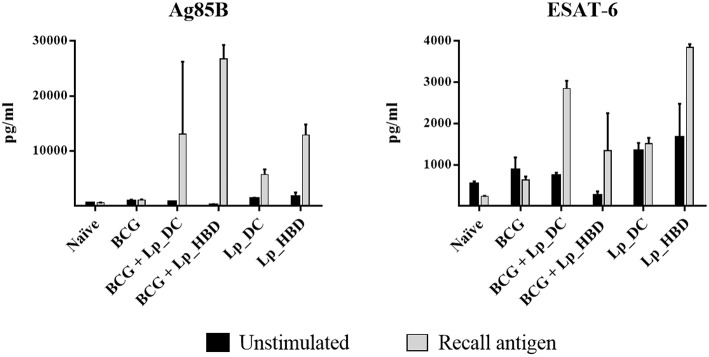
IFN-γ secretion induced by recall antigens in proliferating splenic T cells. Splenocytes from immunized mice were stimulated with Ag85B or ESAT-6 for 6 days and levels of IFN-γ were quantified by ELISA. Results are presented as a mean ± SEM of technical duplicates and are from pooled spleens (*n* = 2–3).

IFN-γ is a Th1 response cytokine playing essential role in protection against intracellular pathogens, such as *M. tuberculosis*, and is generally used as a relevant marker for testing the immunogenic potential of new tuberculosis vaccine candidates (49, 50). We investigated specific IFN-γ responses in proliferating splenic T cells and observed that both Ag85B and ESAT-6 antigens induced IFN-γ secretion for mice immunized with *Lp*_DC or *Lp*_HBD, in both regimes, indicating immunogenicity of *Lp*-based vaccines (Figure 7). No increase in IFN-γ concentrations were detected in cells from the BCG control group (Figure 7).

### *Lp*_DC and *Lp*_HBD Elevate the Frequency of Multifunctional T Cells

Polyfunctional T cells producing multiple pro-inflammatory cytokines, i.e., IFN-γ, TNF-α and IL-2, have been associated with protective immunity against *M. tuberculosis* infections in multiple human and animal studies (51). In this regard, we analyzed quality of CD4^+^ and CD8^+^ T cells from vaccinated mice in terms of IFN-γ, IL-2, TNF-α, and IL-17. We included IL-17, since this cytokine plays an important role in promoting immunity during *M. tuberculosis* infection (52, 53) and is suggested to play a role in vaccine-induced immunity (54).

**Figure 8:**
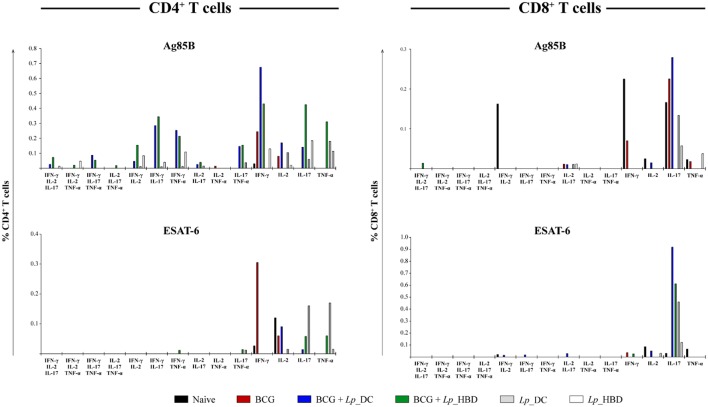
Frequency of CD4^+^ and CD8^+^ T cells producing multiple pro-inflammatory cytokines. Splenocytes from immunized mice were stimulated with Ag85B or ESAT-6 in technical duplicates for 4 h. Splenic T cells producing IFN-γ, IL-2, IL-17, and TNF-α cytokines were determined by intracellular staining using live → single cells → CD3^+^ → CD4^+^/CD8^+^ → IFN-γ^+^/IL-2^+^/IL-17^+^/TNF-α^+^ gating strategy. Thus, the frequencies of single-, double-, and triple- positive CD4^+^ and CD8^+^ T cells were determined. The data are presented from *n* = 2–3 pooled spleens per group.

The results for splenocytes stimulated with Ag85B showed that heterologous immunization led to markedly increased levels of double- and triple-positive cytokine-producing CD4^+^ T cells in mice given the *Lp*_DC construct (Figure 8). When applied in the homologous regimen, *Lp*_DC was able to induce various combinations of double-positive cytokine-producing CD4^+^ T cells (except IL-2^+^TNF-α), whereas no cells producing three cytokines were determined. On the other hand, in the homologous regimen, *Lp*_HBD generated some populations of triple- (IFN-γ^+^IL-2^+^TNF-α^+^ and IFN-γ^+^IL-2^+^IL-17^+^) and double-cytokine-positive CD4^+^ T cells (Figure 8). CD8^+^ T cells producing more than one cytokine were predominantly an IL-2^+^IL-17^+^ population in mice given *Lp*_DC and *Lp*_HBD in both regimens.

Multi-positive T cells recalled by the ESAT-6 antigen were detected to a much lesser degree and comprised some double-positive CD4^+^ T cells in mice vaccinated with *Lp*_HBD in the heterologous (IFN-γ^+^TNF-α^+^ and IL-17^+^TNF-α^+^) or with *Lp*_DC in homologous regimen (IL-17^+^TNF-α^+^), as well as CD8^+^ T cells in mice vaccinated with *Lp*_DC in the heterologous regimen (IFN-γ^+^IL-2^+^, IFN-γ^+^IL-17^+^ and IL-2^+^IL-17^+^) (Figure 8).

### Heterologous Boosting With *Lp* Vaccines Confers Reduction of *M. tuberculosis* Infection in Lungs

We evaluated the protective efficacy of *Lp*-based vaccine candidates in a pathogenic *M. tuberculosis* challenge mouse model. Three weeks after the last immunization mice were infected with aerosolized *M. tuberculosis* (Figure 2) and the bacterial loads in spleens and lungs were determined 4 weeks later. Two independent experiments were performed using the identical immunization regimen and, since animals displayed very similar bacterial loads in their lungs in both experiments for the control (mean CFU_log10_ ± SEM = 6.24 ± 0.07 and 6.17 ± 0.11; *n* = 7) and BCG-immunized (mean CFU_log10_ ± SEM = 5.25 ± 0.06 and 5.19 ± 0.16; *n* = 6-7) groups, we combined the data to increase the statistical power of the assay.

**Figure 9:**
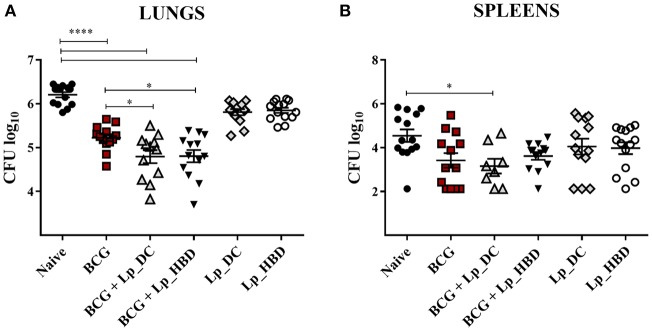
Mycobacterial loads in lungs **(A)** and spleens **(B)** of mice immunized with *Lp* vaccines. Mice were administered with *Lp*_DC or *Lp*_HBD in a heterologous or a homologous immunization regimen, as shown in Figure 2. Three weeks after the last vaccination, mice were infected with *M. tuberculosis* and 4 weeks later bacterial burdens were quantified in lungs and spleens. Results are presented as a cumulative data from two independent experiments and are shown as a mean ± SEM (*n* = 12-14). Statistically significant differences were determined using one-way ANOVA with Tukey's *post-hoc* test and are indicated as follows: *p < 0.05 and ****p < 0.0001.

As expected, parenteral BCG vaccination reduced the mycobacterial burden in lungs and spleens in comparison to naïve mice (Figure 9). Notably, heterologous immunization with both *Lp*_DC and *Lp*_HBD induced statistically significant reduction in *M. tuberculosis* CFU numbers in lungs, when compared to the BCG control group (Figure 9A), indicating a protective potential of the *Lp* constructs when used as a BCG boost. When given as homologous vaccines, both *Lp* candidates showed tendency to lower the mycobacterial burden in lung tissue compared to naïve mice, although insignificantly and not as much as BCG (Figure 9A).

The mycobacterial loads in spleens showed significant reduction of the *M. tuberculosis* load for mice heterologously boosted with *Lp*_DC compared to naïve mice, but there were no significant improvements for the groups that were heterologously boosted with *Lp*_DC or *Lp*_HBD in caparison to BCG-vaccinated mice (Figure 9B). Mice that were not given BCG, but only *Lp*_DC or *Lp*_HBD, displayed a trend for lower mycobacterial loads in spleens similar to BCG. Nevertheless, comparing to the control group, the differences were not statistically significant due to large variations between the individual animals (Figure 9B). Taken together the observations from the pathogenic challenge study indicate that vaccination with *Lp*-based constructs improved the protection afforded by BCG in the lungs and may afford some protection in the lungs and the spleens when used as a homologous vaccination regimen.

## Dicussion

TB still kills millions of people in low- and middle-income countries and the necessity for emerging new vaccines that protect against *M. tuberculosis* infections remains indisputable. TB vaccine development follows two major strategies, both aiming to ensure life-long protection in immunized children: (i) supplement the existing BCG vaccine or (ii) replace BCG by a novel vaccine (55). In the present study we tested two *L. plantarum*-based vaccine candidates in both approaches, namely as BCG booster or primary vaccines.

Based on previous observations suggesting the potential of using *L. plantarum* for vaccine delivery (25, 43), *Lp* was engineered for producing the Ag85B-ESAT-6 fusion antigen and to target this antigen to the bacterial surface. The antigen was C-terminally extended by a DC-binding peptide (40, 41) or an HBD-domain (45–47), resulted in two vaccine constructs: *Lp*_DC and *Lp*_HBD, respectively (Figure 1).

We had earlier shown that *Lp*_DC induced TB-specific immune responses when given through mucosal routes (25). The goal of the present study was to investigate the protective efficacy of the *Lp*-based TB vaccine candidates and to analyze specific parameters that might correlate with protection. The *Lp*_HBD construct was generated because the HBHA adhesin is a known important virulence factor of *M. tuberculosis* that binds epithelial cells and plays a crucial role in extrapulmonary dissemination of the pathogen (56–58). Figure 3 shows that this new antigen construct was successfully produced and target to the bacterial surface.

*Lactobacillus plantarum* has been shown to activate innate immune cells and skew the immune system toward Th1 responses (35, 36). Indeed, we found that human and mouse APCs pulsed with *Lp*_DC or *Lp*_HBD expressed important co-stimulatory molecules, such as CD80, CD40, CD86, and MHC class I/II (Figure 4), which are indicative of maturation processes that are essential for triggering secondary immune responses and post-immunization T cell activation (3, 48, 59). We observed down-regulation of MHC class II in *Lp*-stimulated murine DCs cells (Figure 4B) and that phenomenon has been observed before in studies with bone-marrow derived DCs (60). We quantified expression of PD-L1 and PD-L2, because there is evidence that these molecules regulate T cells function (61). Both mouse macrophages and DCs expressed PD-L1 (Figures 4B,C), while PD-L2 was expressed by macrophages but down-regulated in DCs (Figure 4B). Although regulation of PD-L1 and PD-L2 may differ (62), we cannot presently explain the observed down-regulation in murine DCs. The expression of chemokine receptor CCR7 may be crucial for migration of activated DCs to lung-draining lymph nodes; however, CCR7 expression by mouse DCs stimulated with *Lp* constructs was low (Figure 4B). It is important to note that recent evidence has highlighted that GM-CSF-generated BMDCs contain macrophages as well as DCs (63). Therefore, we cannot exclude the possibility of non-DCs responding to our vaccine constructs. Future work will specifically explore the role of various DC and macrophage subsets in the immunological mechanisms of vaccine efficacy.

*Lp* constructs were given to mice as an intranasal booster to BCG or as a primary vaccine administered first parenterally followed by intranasal boosts (Figure 2). Although protective, BCG is considered a rather poor inducer of antibody or T cells responses (3, 64) and that trend was also observed in this study. We investigated humoral and cellular immune responses induced by *Lp*_DC and *Lp*_HBD and further evaluated in what degree the immunogenic determinants corresponded to observed protective effects. Humoral immunity induced by *Lp*-based vaccines was generally poor and was mainly manifested in increased IgA levels in lungs of mice that had received *Lp*_DC in heterologous regimen (Figure 5). It has been considered for years that the contribution of humoral immunity in controlling *M. tuberculosis* infections is limited. Nowadays however, there is evidence indicating that antibody-mediated responses may restrict pathogen dissemination and play a significant role in protection (6, 65). In contrast, the prominence of cellular immunity is widely recognized (6), and T cells are considered as major determinants of protection against *M. tuberculosis* infections. The heterologous vaccination regimen applied in the current study led to increased levels of CD4^+^ and CD8^+^ T cells, while this was not the case when using *Lp* constructs as primary vaccines (Figure 6). Effective vaccine is intended to establish long-lived population of memory T cells (66) and, notably, BCG boost with both *Lp*_DC and *Lp*_HBD constructs generated levels of memory cells among proliferative T cells, with the effector memory cells (T_EM_) as the major populations (Figure 6), which has been observed in previous studies (67, 68).

Another important parameter of TB vaccine potential is IFN-γ production by T cells, since this cytokine is known to maintain cellular Th1 responses and to activate macrophages controlling intracellular pathogens such as *M. tuberculosis* (69, 70). We measured elevated INF-γ secretion by splenic cells stimulated with Ag85B and to a lesser extent ESAT-6, for all groups vaccinated with *Lp* constructs and in both regimens (Figure 7). Somewhat unexpectedly, INF-γ production by splenic T cells from mice that had received *Lp*_DC or *Lp*_HBD as the primary vaccine was not reflected in increased generation of memory T cells (Figure 6). This discrepancy can be explained if one assumes that T cells producing only INF-γ are restricted in becoming memory cells and that, rather multifunctional T cells, expressing a combination of IFN- γ, IL-2 and TNF-α, are more prone to become memory cells (66, 71). This assumption was supported by the work of Aagaard et al., who demonstrated that increases in double- (TNF-α and IL-2) or triple-positive (IFN-γ, IL-2 and TNF-α) CD4^+^ T cells correlated with protection offered by TB vaccine candidates in a mouse model (72). While we detected clear increases in Ag85B-recalled double- and triple- positive CD4^+^ T cells from mice immunized with *Lp* constructs in the heterologous regimen, these increases were much less prominent for groups given *Lp* as a primary vaccine (Figure 8), in seeming accordance with lower levels of memory T cells. It should be noted though that, although the role of multifunctional CD4^+^ in protective immunity has been emphasized in numerous studies, more recent evidence indicates that polyfunctional CD4^+^ T cells might be insufficient or even non-associated with protection in TB vaccine development (51).

While the multifunctional CD4^+^ T cells are widely studied, an impact of multifunctional CD8^+^ T cells in protective immunity remains little discussed in literature. Nonetheless, multifunctional CD8^+^ T cells (IFN-γ, TNF-α, and IL-2) have been linked to reduced risk of reactivation and enhanced control of active TB in humans (73). In our study, immunization with *Lp*_DC or *Lp*_HBD did not induce any triple-positive CD8^+^ T cell phenotypes and only low frequencies of a few double-functional T cells (Figure 8).

Vaccine-induced IL-17 production in lungs has been shown to be crucial for induction of T cell chemokines and recruitment of CD4^+^ T cells after aerosol *M. tuberculosis* challenge (74). Interestingly, we detected an elevated fraction of IL-17-producing CD4^+^ T cells specific to mycobacterial antigens in all groups given *Lp*, but not in the BCG control group (Figure 8). This finding further supports the beneficial effect of using *Lp*-based TB vaccines rather than BCG alone, which was also reflected in increased protection in lungs when the heterologous regimen was applied.

The most important finding of this study is that intranasally administered *Lp*_DC and *Lp*_HBD enhanced the protection provided by BCG in terms of reduced *M. tuberculosis* CFU in lungs. When used as primary vaccines, the *Lp* constructs tended to reduce bacterial load in lungs (comparing to non-immunized mice), but effects were insignificant and not as good as for BCG. One possible explanation for the lesser efficiency of the homologous regimen can be the superior immunogenic and antigenic nature of BCG over *Lp*. BCG is an attenuated pathogenic bacterium, rich in strong mycobacterial antigens and PAMPs, while *Lp* constructs remain non-pathogenic bacteria expressing two antigens of *M. tuberculosis*. It cannot be excluded that better results may be obtained when using *Lp* vaccines in a homologous regimen, for example by optimizing the numbers of administered *Lp* cells. The CFU data for spleens did show lower CFU numbers in all vaccinated groups in relation to naïve mice, but did not show statistically significant effects of the *Lp* constructs in comparison to BCG-vaccinated mice. It is therefore possible that the protective effect of the *Lp* constructs is smaller once the pathogen has disseminated from lungs.

The measured protective immunity in lungs correlated with cellular responses (i.e., proliferative responses, high IFN-γ levels and high frequency of polyfunctional T cells), as well as the modest elevation of lung IgA, induced by *Lp*_DC and *Lp*_HBD in the heterologous regimen.

We used a DC-targeting peptide because our own unpublished results had shown that this is beneficial for immunogenicity and because the peptide was proven as an essential element of an oral vaccine against anthrax (41). The present data show that the two investigated constructs, *Lp*_DC and *Lp*_HBD, are quite similar in terms of potential for protection, and the ability to activate APCs and evoke cellular immunity. It would be of interest to further investigate the importance and the (possible) functional differences of the two targeting sequences (DC-targeting peptide and HBD) exploited in this study.

*Lactobacillus plantarum* has been presented as a potential candidate for mucosal vaccine against tuberculosis that induced a favorable immunogenic profile previously (25, 75). Yet, to our knowledge, increased protection against *M. tuberculosis* infection evoked by an engineered *Lactobacillus* has not been reported before.

Taken together, this study shows that, when used as an intranasal booster vaccine, two *Lp*-based constructs, *Lp*_DC and *Lp*_HBD, triggered diverse components of immune system and enhanced protection conferred by BCG. Primary immunization with the *Lp* vaccine candidates induced some protective immunity, but the effects were not as robust as for single parenteral BCG vaccination. Thus, *Lp*_DC and *Lp*_HBD have potential as a mucosal booster vaccine to BCG and merit further development as a novel TB vaccine platform.

## Data Availability

The raw data supporting the conclusions of this manuscript will be made available by the authors, without undue reservation, to any qualified researcher.

## Ethics Statement

Human peripheral blood mononuclear cells were isolated and handled according to institutional ethical guidelines (Østfold Hospital Trust, Norway). All animals were used with approval from St. George's University of London Ethics Committee under an approved UK Home Office animal project license and used in accordance with the Animals (Scientific Procedures) Act 1986.

## Author Contributions

KK, AC, GM, VE, and RR conceived and planned the experiments. KK, LØ, AT, AC, MP, and RR carried out all the laboratory experiments. KK, AC, MP, and RR contributed to the result analysis. KK drafted the paper. VE, GM, and RR contributed to preparing the final version of the manuscript. All authors read and approved the final manuscript.

### Conflict of Interest Statement

The authors declare that the research was conducted in the absence of any commercial or financial relationships that could be construed as a potential conflict of interest.

## References

[1] Evans TG Schrager L Thole J. Status of vaccine research and development of vaccines for tuberculosis. Vaccine. (2016) 34:2911–4. 10.1016/j.vaccine.2016.02.079 26973073

[2] Who. Global Tuberculosis Report 2018. World Health Organization (2018).

[3] Copland A Diogo GR Hart P Harris S Tran AC Paul MJ. Mucosal delivery of fusion proteins with *Bacillus subtilis* spores enhances protection against tuberculosis by Bacillus Calmette-Guérin. Front Immunol. (2018) 9:346. 10.3389/fimmu.2018.00346 29593708 PMC5857916

[4] Kaufmann SH. Novel tuberculosis vaccination strategies based on understanding the immune response. J Intern Med. (2010) 267:337–53. 10.1111/j.1365-2796.2010.02216.x 20433580

[5] Luca S Mihaescu T. History of BCG vaccine. Mædica. (2013) 8:53–8. 24023600 PMC3749764

[6] Fletcher HA Schrager L. TB vaccine development and the end TB strategy: importance and current status. Trans R Soc Trop Med Hyg. (2016) 110:212–8. 10.1093/trstmh/trw016 27076508 PMC4830404

[7] Moliva JI Turner J Torrelles JB. Prospects in *Mycobacterium bovis* Bacille Calmette et Guérin (BCG) vaccine diversity and delivery: why does BCG fail to protect against tuberculosis? Vaccine. (2015) 33:5035–41. 10.1016/j.vaccine.2015.08.033 26319069 PMC4577463

[8] Hesseling AC Marais BJ Gie RP Schaaf HS Fine PEM Godfrey-Faussett P. The risk of disseminated Bacille Calmette-Guerin (BCG) disease in HIV-infected children. Vaccine. (2007) 25:14–8. 10.1016/j.vaccine.2006.07.020 16959383

[9] Kaufmann SHE Weiner J Von Reyn CF. Novel approaches to tuberculosis vaccine development. Int J Infect Dis. (2017) 56:263–7. 10.1016/j.ijid.2016.10.018 27816661

[10] Ottenhoff THM Kaufmann SHE. Vaccines against tuberculosis: where are we and where do we need to go? PLoS Pathog. (2012) 8:e1002607. 10.1371/journal.ppat.1002607 22589713 PMC3349743

[11] Beverley PCL Sridhar S Lalvani A Tchilian EZ. Harnessing local and systemic immunity for vaccines against tuberculosis. Mucosal Immunol. (2013) 7:20. 10.1038/mi.2013.99 24253104

[12] Bull NC Stylianou E Kaveh DA Pinpathomrat N Pasricha J Harrington-Kandt R. Enhanced protection conferred by mucosal BCG vaccination associates with presence of antigen-specific lung tissue-resident PD-1+ KLRG1– CD4+ T cells. Mucosal Immunol. (2019) 12:555–64. 10.1038/s41385-018-0109-1 30446726 PMC7051908

[13] Stylianou E Griffiths KL Poyntz HC Harrington-Kandt R Dicks MD Stockdale L. Improvement of BCG protective efficacy with a novel chimpanzee adenovirus and a modified vaccinia Ankara virus both expressing Ag85A. Vaccine. (2015) 33:6800–8. 10.1016/j.vaccine.2015.10.017 26478198 PMC4678294

[14] Tameris MD Hatherill M Landry BS Scriba TJ Snowden MA Lockhart S. Safety and efficacy of MVA85A, a new tuberculosis vaccine, in infants previously vaccinated with BCG: a randomised, placebo-controlled phase 2b trial. Lancet. (2013) 381:1021–8. 10.1016/S0140-6736(13)60177-4 23391465 PMC5424647

[15] Smaill F Xing Z. Human type 5 adenovirus-based tuberculosis vaccine: is the respiratory route of delivery the future? Expert Rev Vaccines. (2014) 13:927–30. 10.1586/14760584.2014.929947 24935214

[16] Walker KB Guo M Guo Y Poecheim J Velmurugan K Schrager LK. Novel approaches to preclinical research and TB vaccine development. Tuberculosis. (2016) 99:S12–5. 10.1016/j.tube.2016.05.012 27452413

[17] Elamin AA Stehr M Spallek R Rohde M Singh M. The *Mycobacterium tuberculosis* Ag85A is a novel diacylglycerol acyltransferase involved in lipid body formation. Mol Microbiol. (2011) 81:1577–92. 10.1111/j.1365-2958.2011.07792.x 21819455

[18] Jia Q Dillon BJ Maslesa-Galic S Horwitz MA Listeria-vectored vaccine expressing the *Mycobacterium tuberculosis* 30 kDa major secretory protein via the constitutively active *prf* A^*^ regulon boosts BCG efficacy against tuberculosis. Infect Immun. (2017) 85:e00245–17. 10.1128/IAI.00245-17 28630063 PMC5563566

[19] Yin Y Lian K Zhao D Tao C Chen X Tan W. A promising *Listeria*-vectored vaccine induces Th1-type immune responses and confers protection against tuberculosis. Front Cell Infect Microbiol. (2017) 7:407. 10.3389/fcimb.2017.00407 29034213 PMC5626977

[20] Mollenkopf H-J Groine-Triebkorn D Andersen P Hess J Kaufmann SHE. Protective efficacy against tuberculosis of ESAT-6 secreted by a live *Salmonella typhimurium* vaccine carrier strain and expressed by naked DNA. Vaccine. (2001) 19:4028–35. 10.1016/S0264-410X(01)00109-8 11427279

[21] Parida SK Huygen K Ryffel B Chakraborty T. Novel bacterial delivery system with attenuated *Salmonella typhimurium* carrying plasmid encoding *Mtb* Antigen 85A for mucosal ommunization: establishment of proof of principle in TB mouse model. Ann N Y Acad Sci. (2005) 1056:366–78. 10.1196/annals.1352.030 16387702

[22] Das K Thomas T Garnica O Dhandayuthapani S Recombinant *Bacillus subtilis* spores for the delivery of *Mycobacterium tuberculosis* Ag85B-CFP10 secretory antigens. Tuberculosis. (2016) 101:S18–27. 10.1016/j.tube.2016.09.016 27727129

[23] Sibley L Reljic R Radford DS Huang J-M Hong HA Cranenburgh RM. Recombinant *Bacillus subtilis* spores expressing MPT64 evaluated as a vaccine against tuberculosis in the murine model. FEMS Microbiol Lett. (2014) 358:170–9. 10.1111/1574-6968.12525 24990572

[24] Bermudez-Humaran LG Kharrat P Chatel JM Langella P. Lactococci and lactobacilli as mucosal delivery vectors for therapeutic proteins and DNA vaccines. Microb Cell Fact. (2011) 10:S4. 10.1186/1475-2859-10-S1-S4 21995317 PMC3231930

[25] Kuczkowska K Kleiveland CR Minic R Moen LF Øverland L Tjåland R. Immunogenic properties of *Lactobacillus plantarum* producing surface-displayed *Mycobacterium tuberculosis* antigens. Appl Environ Microbiol. (2017) 83:e02782–16. 10.1128/AEM.02782-16 27815271 PMC5203620

[26] Leblanc JG Aubry C Cortes-Perez NG De Moreno De Leblanc A Vergnolle N Langella P. Mucosal targeting of therapeutic molecules using genetically modified lactic acid bacteria: an update. FEMS Microbiol Lett. (2013) 344:1–9. 10.1111/1574-6968.12159 23600579

[27] Wyszyńska A Kobierecka P Bardowski J Jagusztyn-Krynicka E Lactic acid bacteria—20 years exploring their potential as live vectors for mucosal vaccination. Appl Microbiol Biotechnol. (2015) 99:2967–77. 10.1007/s00253-015-6498-0 25750046 PMC4365182

[28] Herrera Ramírez JC De La Mora AC De La Mora Valle A Lopez-Valencia G Hurtado RMB Rentería Evangelista TB Immunopathological evaluation of recombinant mycobacterial antigen Hsp65 expressed in *Lactococcus lactis* as a novel vaccine candidate. Infect Immun. (2017) 18:197–202. PMC5674443 29163649

[29] Mancha-Agresti P De Castro CP Dos Santos JSC Araujo MA Pereira VB Leblanc JG. Recombinant invasive *Lactococcus lactis* carrying a DNA vaccine coding the Ag85A antigen increases INF-γ, IL-6, and TNF-α cytokines after intranasal immunization. Front Microbiol. (2017) 8:1263. 10.3389/fmicb.2017.01263 28744263 PMC5504179

[30] Pereira VB Cunha VP Preisser TM Souza BM Turk MZ De Castro CP. *Lactococcus lactis* carrying a DNA vaccine coding for the ESAT-6 antigen increases IL-17 cytokine secretion and boosts the BCG vaccine immune response. J Appl Microbiol. (2017) 122:1657–62. 10.1111/jam.13449 28314076

[31] Pereira VB Saraiva TD Souza BM Zurita-Turk M Azevedo MS De Castro CP. Development of a new DNA vaccine based on mycobacterial ESAT-6 antigen delivered by recombinant invasive *Lactococcus lactis* FnBPA+. Appl Microbiol Biotechnol. (2015) 99:1817–26. 10.1007/s00253-014-6285-3 25503506

[32] Baarlen P Troost FJ Hemert S Meer C Vos WM Groot PJ. Differential NF-kappaB pathways induction by *Lactobacillus plantarum* in the duodenum of healthy humans correlating with immune tolerance. Proc Natl Acad Sci USA. (2009) 106:2371–6. 10.1073/pnas.0809919106 19190178 PMC2650163

[33] Bloksma N De Heer E Van Dijk H Willers J. Adjuvanticity of lactobacilli. I Differential effects of viable and killed bacteria. Clin Exp Immunol. (1979) 37:367. 387312 PMC1537799

[34] Bloksma N Van Dijk H Korst P Willers JM. Cellular and humoral adjuvant activity of a mistletoe extract. Z Immunitatsforsch Immunobio. (1979) 156:309–19. 10.1016/S0340-904X(79)80052-4 575523

[35] De Vries M Vaughan E Kleerebezem M De Vos W *Lactobacillus plantarum*—survival, functional and potential probiotic properties in the human intestinal tract. Int Dairy J. (2006) 16:1018–28. 10.1016/j.idairyj.2005.09.003

[36] Dong H Rowland I Yaqoob P. Comparative effects of six probiotic strains on immune function *in vitro*. Br J Nutr. (2012) 108:459–70. 10.1017/S0007114511005824 22054064

[37] Pouwels PH Leer RJ Boersma WJ. The potential of *Lactobacillus* as a carrier for oral immunization: development and preliminary characterization of vector systems for targeted delivery of antigens. J Biotechnol. (1996) 44:183–92. 10.1016/0168-1656(95)00140-9 8717402

[38] Fredriksen L Kleiveland CR Hult LTO Lea T Nygaard CS Eijsink VG. Surface display of N-terminally anchored invasin by *Lactobacillus plantarum* activates NF-κB in monocytes. Appl Environ Microbiol. (2012) 78:5864–71. 10.1128/AEM.01227-12 22706054 PMC3406107

[39] Kleerebezem M Boekhorst J Kranenburg R Molenaar D Kuipers OP Leer R. Complete genome sequence of *Lactobacillus plantarum* WCFS1. Proc Natl Acad Sci USA. (2003) 100:1880–995. 10.1073/pnas.0337704100 12566566 PMC149946

[40] Curiel TJ Morris C Brumlik M Landry SJ Finstad K Nelson A. Peptides identified through phage display direct immunogenic antigen to dendritic cells. J Immunol. (2004) 172:7425–31. 10.4049/jimmunol.172.12.7425 15187120

[41] Mohamadzadeh M Duong T Sandwick S Hoover T Klaenhammer T. Dendritic cell targeting of *Bacillus anthracis* protective antigen expressed *Lactobacillus acidophilus* protects mice from lethal challenge. Proc Natl Acad Sci USA. (2009) 106:4331–6. 10.1073/pnas.0900029106 19246373 PMC2647975

[42] Aukrust TW Brurberg MB Nes IF. Transformation of *Lactobacillus* by electroporation. In: Nickoloff JA, editor. Electroporation Protocols for Microorganisms. Boston, MA: Springer (1995). p. 201–8. 10.1385/0-89603-310-4:201 7550736

[43] Kuczkowska K Myrbraten I Overland L Eijsink VGH Follmann F Mathiesen G. *Lactobacillus plantarum* producing a *Chlamydia trachomatis* antigen induces a specific IgA response after mucosal booster immunization. PLoS ONE. (2017) 12:e0176401. 10.1371/journal.pone.0176401 28467432 PMC5415134

[44] Inaba K Inaba M Romani N Aya H Deguchi M Ikehara S. Generation of large numbers of dendritic cells from mouse bone marrow cultures supplemented with granulocyte/macrophage colony-stimulating factor. J Exp Med. (1992) 176:1693–702. 10.1084/jem.176.6.1693 1460426 PMC2119469

[45] Delogu G Fadda G Brennan MJ. Impact of structural domains of the heparin binding hemagglutinin of *Mycobacterium tuberculosis* on function. Protein Pept Lett. (2012) 19:1035–9. 10.2174/092986612802762697 22533618

[46] Delogu G Sanguinetti M Posteraro B Rocca S Zanetti S Fadda G. The *hbha* gene of *Mycobacterium tuberculosis* is specifically upregulated in the lungs but not in the spleens of aerogenically infected mice. Infect Immun. (2006) 74:3006–11. 10.1128/IAI.74.5.3006-3011.2006 16622240 PMC1459695

[47] Esposito C Marasco D Delogu G Pedone E Berisio R. Heparin-binding hemagglutinin HBHA from *Mycobacterium tuberculosis* affects actin polymerisation. Biochem Biophys Res Commun. (2011) 410:339–44. 10.1016/j.bbrc.2011.05.159 21672524

[48] Storni T Lechner F Erdmann I Bächi T Jegerlehner A Dumrese T. Critical role for activation of antigen-presenting cells in priming of cytotoxic T cell responses after vaccination with virus-like particles. J Immunol. (2002) 168:2880–6. 10.4049/jimmunol.168.6.2880 11884458

[49] Agger EM Andersen P. Tuberculosis subunit vaccine development: on the role of interferon-γ. Vaccine. (2001) 19:2298–302. 10.1016/S0264-410X(00)00519-3 11257351

[50] Lalvani A Millington KA. T cells and tuberculosis: beyond interferon-γ. J Infect Dis. (2008) 197:941–3. 10.1086/529049 18419531

[51] Lewinsohn DA Lewinsohn DM Scriba TJ. Polyfunctional CD4(+) T cells as targets for tuberculosis vaccination. Front Immunol. (2017) 8:1262. 10.3389/fimmu.2017.01262 29051764 PMC5633696

[52] Coulter F Parrish A Manning D Kampmann B Mendy J Garand M. IL-17 production from T helper 17, mucosal-associated invariant T, and γ*δ* cells in tuberculosis infection and disease. Front Immunol. (2017) 8:1252. 10.3389/fimmu.2017.01252 29075255 PMC5641565

[53] Torrado E Cooper AM. IL-17 and Th17 cells in tuberculosis. Cytokine Growth Factor Rev. (2010) 21:455–62. 10.1016/j.cytogfr.2010.10.004 21075039 PMC3032416

[54] Lin Y Slight SR Khader SA. Th17 cytokines and vaccine induced immunity. Semin Immunopathol. (2010) 32:79–90. 10.1007/s00281-009-0191-2 20112107 PMC2855296

[55] Mcshane H Williams A. A review of preclinical animal models utilised for TB vaccine evaluation in the context of recent human efficacy data. Tuberculosis. (2014) 94:105–10. 10.1016/j.tube.2013.11.003 24369986 PMC3969587

[56] Pethe K Alonso S Biet F Delogu G Brennan MJ Locht C. The heparin-binding haemagglutinin of *M. tuberculosis* is required for extrapulmonary dissemination. Nature. (2001) 412:190–4. 10.1038/35084083 11449276

[57] Pethe K Aumercier M Fort E Gatot C Locht C Menozzi FD. Characterization of the heparin-binding site of the mycobacterial heparin-binding hemagglutinin adhesin. J Biol Chem. (2000) 275:14273–80. 10.1074/jbc.275.19.14273 10799506

[58] Vidal Pessolani MC Marques MA Reddy VM Locht C Menozzi FD. Systemic dissemination in tuberculosis and leprosy: do mycobacterial adhesins play a role? Microbes Infect. (2003) 5:677–84. 10.1016/S1286-4579(03)00098-4 12787744

[59] Saban DR. The chemokine receptor CCR7 expressed by dendritic cells: A key player in corneal and ocular surface inflammation. Ocular Surface. (2014) 12:87–99. 10.1016/j.jtos.2013.10.007 24725321 PMC3986807

[60] Villadangos JA Cardoso MA Steptoe RJ Van Berkel D Pooley J Carbone FR. MHC class II expression is regulated in dendritic cells independently of invariant chain degradation. Immunity. (2001) 14:739–49. 10.1016/S1074-7613(01)00148-0 11420044

[61] Karunarathne DS Horne-Debets JM Huang JX Faleiro R Leow CY Amante F. Programmed death-1 ligand 2-mediated regulation of the PD-L1 to PD-1 axis is essential for establishing CD4(+) T cell immunity. Immunity. (2016) 45:333–45. 10.1016/j.immuni.2016.07.017 27533014

[62] Loke PN Allison JP. PD-L1 and PD-L2 are differentially regulated by Th1 and Th2 cells. Proc Natl Acad Sci USA. (2003) 100:5336–41. 10.1073/pnas.0931259100 12697896 PMC154346

[63] Helft J Bottcher J Chakravarty P Zelenay S Huotari J Schraml BU. GM-CSF mouse bone marrow cultures comprise a heterogeneous population of CD11c(+)MHCII(+) macrophages and dendritic cells. Immunity. (2015) 42:1197–211. 10.1016/j.immuni.2015.05.018 26084029

[64] Mittrücker H-W Steinhoff U Köhler A Krause M Lazar D Mex P. Poor correlation between BCG vaccination-induced T cell responses and protection against tuberculosis. Proc Natl Acad Sci USA. (2007) 104:12434–9. 10.1073/pnas.0703510104 17640915 PMC1941486

[65] Jacobs AJ Mongkolsapaya J Screaton GR Mcshane H Wilkinson RJ. Antibodies and tuberculosis. Tuberculosis. (2016) 101:102–13. 10.1016/j.tube.2016.08.001 27865379 PMC5120988

[66] Henao-Tamayo M Ordway DJ Orme IM. Memory T cell subsets in tuberculosis: what should we be targeting? Tuberculosis. (2014) 94:455–61. 10.1016/j.tube.2014.05.001 24993316

[67] El Fenniri L Toossi Z Aung H El Iraki G Bourkkadi J Benamor J. Polyfunctional *Mycobacterium tuberculosi*s-specific effector memory CD4+ T cells at sites of pleural TB. Tuberculosis. (2011) 91:224–30. 10.1016/j.tube.2010.12.005 21459675 PMC3306579

[68] Purwar R Campbell J Murphy G Richards WG Clark RA Kupper TS. Resident memory T cells (T(RM)) are abundant in human lung: diversity, function, and antigen specificity. PLoS ONE. (2011) 6:e16245. 10.1371/journal.pone.0016245 21298112 PMC3027667

[69] Bhatt K Verma S Ellner JJ Salgame P. Quest for correlates of protection against tuberculosis. Clin Vaccine Immunol. (2015) 22:258–66. 10.1128/CVI.00721-14 25589549 PMC4340894

[70] Flynn JL Chan J Triebold KJ Dalton DK Stewart TA Bloom BR. An essential role for interferon gamma in resistance to *Mycobacterium tuberculosis* infection. J Exp Med. (1993) 178:2249–54. 10.1084/jem.178.6.2249 7504064 PMC2191274

[71] Seder RA Darrah PA Roederer M. T-cell quality in memory and protection: implications for vaccine design. Nat Rev Immunol. (2008) 8:247–58. 10.1038/nri2274 18323851

[72] Aagaard CS Hoang TT Vingsbo-Lundberg C Dietrich J Andersen P. Quality and vaccine efficacy of CD4+ T cell responses directed to dominant and subdominant epitopes in ESAT-6 from *Mycobacterium tuberculosis*. J Immunol. (2009) 183:2659–68. 10.4049/jimmunol.0900947 19620314

[73] Day CL Abrahams DA Lerumo L Janse Van Rensburg E Stone L O'rie T. Functional capacity of *Mycobacterium tuberculosis*-specific T cell responses in humans is associated with mycobacterial load. J Immunol. (2011) 187:2222–32. 10.4049/jimmunol.1101122 21775682 PMC3159795

[74] Khader SA Bell GK Pearl JE Fountain JJ Rangel-Moreno J Cilley GE. IL-23 and IL-17 in the establishment of protective pulmonary CD4+ T cell responses after vaccination and during *Mycobacterium tuberculosis* challenge. Nat Immunol. (2007) 8:369. 10.1038/ni1449 17351619

[75] Mustafa AD Kalyanasundram J Sabidi S Song AAL Abdullah M. Proof of concept in utilizing in-trans surface display system of *Lactobacillus plantarum* as mucosal tuberculosis vaccine via oral administration in mice. BMC Biotechnol. (2018) 18:63. 10.1186/s12896-018-0461-y 30309359 PMC6182793

